# Refining the safety assessment of newly expressed proteins in GMOs

**DOI:** 10.3389/ftox.2025.1679506

**Published:** 2025-10-13

**Authors:** Elena Sánchez-Brunete, Arianna Ferrari, F. Javier Moreno, Tilemachos Goumperis, Michele Ardizzone, Reinhilde Schoonjans, Ian Dewhurst, Ivan Dimitrov, Michelle M. Epstein, Frits Koning, Kevin Hogeveen, Antonio Fernández-Dumont

**Affiliations:** ^1^ European Food Safety Authority (EFSA), Parma, Italy; ^2^ Instituto de Investigación en Ciencias de la Alimentación, CIAL (CSIC-UAM), Madrid, Spain; ^3^ Formerly at Chemicals Regulation Division (CRD), York, United Kingdom; ^4^ Medical University of Sofia, Sofia, Bulgaria; ^5^ Laboratory of Experimental Allergy, Department of Dermatology, Medical University of Vienna, Vienna, Austria; ^6^ Leiden University Medical Center, Leiden, Netherlands; ^7^ Agence nationale de sécurité sanitaire de l’alimentation, de l’environnement et du travail (ANSES), Maisons Alfort, France

**Keywords:** protein safety, newly expressed proteins, GMO, risk assessment, new approach methodologies, 3Rs

## Abstract

The European Food Safety Authority (EFSA) carries out safety assessments of newly expressed proteins (NEPs) in genetically modified organisms (GMOs). Here, toxicity and allergenicity assessments are the cornerstone of NEP evaluation, ensuring that any potential health hazards are rigorously identified and characterised. Recent examples of EFSA’s NEP safety assessments illustrate how novel methodologies, alongside established ones reconsidered from new perspectives, guide case-by-case decisions. These advances provide an opportunity to improve the robustness, proportionality, and scientific credibility of risk assessments. Moreover, it may alleviate the need for *in vivo* animal testing. Building on this development, EFSA aims to integrate new approach methodologies (NAMs) into risk assessment to provide a scientific basis for waiving *in vivo* testing, aligning its approach with the 3Rs principles (Replacement, Reduction, Refinement) and the European Commission’s roadmap for phasing out animal testing. Overall, this shift reflects a broader transformation in EFSA’s safety assessment of NEPs, characterised by openness to innovation, optimisation of existing methods, and ensuring preparedness for future risk assessment needs. The ultimate goal is to ensure the highest level of protection for human and animal health, while embracing scientific progress.

## 1 Introduction

Genetically modified organisms (GMOs) must undergo rigorous food, feed, and environmental safety assessments by regulatory authorities in relevant jurisdictions worldwide to obtain market authorisation. A wide range of GMOs have been approved for commercial use in numerous countries (ISAAA, 2025). In the European Union (EU), GMO risk assessments are conducted by the European Food Safety Authority (EFSA) (EFSA, 2025) and has led to the market authorisation of over 100 GMOs (European Commission, 2025a).

The key component of the risk assessment of GMOs involves evaluating the safety of newly expressed proteins (NEPs) introduced through genetic modification. The main strategy for NEP safety assessment in GMOs are rooted in chemical risk assessment principles and the *Codex Alimentarius* guidelines for biotechnology-derived foods, firstly published in 2003 (Codex Alimentarius, 2003–2009). These guidelines were designed for simpler GMO products expressing a relatively low number of NEPs and for proteins amenable to various testing strategies. However, the risk assessment of GMOs is becoming increasingly complex due to the growing occurrence of NEPs that are difficult to characterise and evaluate using existing methodologies (e.g., membrane-bound proteins, transcription factors), as well as products expressing numerous NEPs. Two decades of experience at EFSA, along with the emergence of new methodologies, have highlighted the need to revise and modernise the conventional approach to NEP safety assessment (EFSA GMO Panel et al., 2025b).

This work outlines the evolution of EFSA’s approach to NEP safety assessment by comparing the traditional framework with the updated strategy currently being integrated. New Approach Methodologies (NAMs), which include *in silico* and *in vitro* methods used as alternatives to animal testing, play a central role in this transition. Although NAMs are already widely applied as research tools, their use in regulatory risk assessments remains limited (Cattaneo et al., 2023). The refined approach aims to strengthen the scientific justification for waiving certain studies, particularly *in vivo* testing and non-targeted *in vitro* assays. Its objectives are: (i) to support the reduction of animal use in line with the 3Rs principles (Replacement, Reduction, and Refinement), (ii) to align with the European Commission’s roadmap for phasing out animal studies (European Commission, 2025b; Walder et al., 2025), and (iii) to promote the development and implementation of more targeted, efficient testing strategies that are proportionate to the actual level of risk identified.

## 2 The evolution of EFSA’s approach to NEP safety assessment in GMOs

The safety evaluation of NEPs includes a case-by-case assessment of potential toxicity and allergenicity, following a weight-of-evidence (WoE) approach. If there is sufficient evidence that the NEP under assessment has a history of safe use (HoSU), *in vitro* studies or animal testing, such as the 28-day repeated-dose study, can be waived (EFSA GMO Panel, 2011; European Commission, 2013). If the HoSU of the NEP cannot be demonstrated, the traditional assessment strategy is based on the integration of data from multiple lines of evidence. This includes the molecular and biochemical characterisation of the NEPs as expressed in the plants, or surrogates, homology searches against known toxic and allergenic proteins, studies on protein stability and the investigation of catalytic properties in the case of enzymes. Additional considerations include resistance to proteolytic enzymes and animal toxicity studies (EFSA GMO Panel, 2011).

The frameworks underpinning the current approach were originally developed to assess safety of small chemical compounds added to or naturally occurring in food and feed. However, proteins differ significantly from such chemicals in many ways, including molecular weight, three-dimensional conformation, chemical composition, digestion and absorption kinetics, and specific biological function. Given these differences and the increasing complexity of some GMOs, there is a need for a more flexible, case-specific approach (Ferrari et al., 2025). EFSA is advancing in this direction by integrating additional technologies and data sources, such as 3D structural similarity analyses, read-across and considerations of HoSU. Such evidence can complement traditional approaches or, when more effective, serve as an alternative with the potential to simplify, adapt, and reinforce the process while still meeting the same data requirements (EFSA GMO Panel et al., 2025b). However, further work is needed to establish consensus and clear criteria for the concept of HoSU. Building international consensus on the HoSU concept has already been identified as a priority (Fernandez and Paoletti, 2018; EFSA GMO Panel et al., 2025b).

**FIGURE 1:**
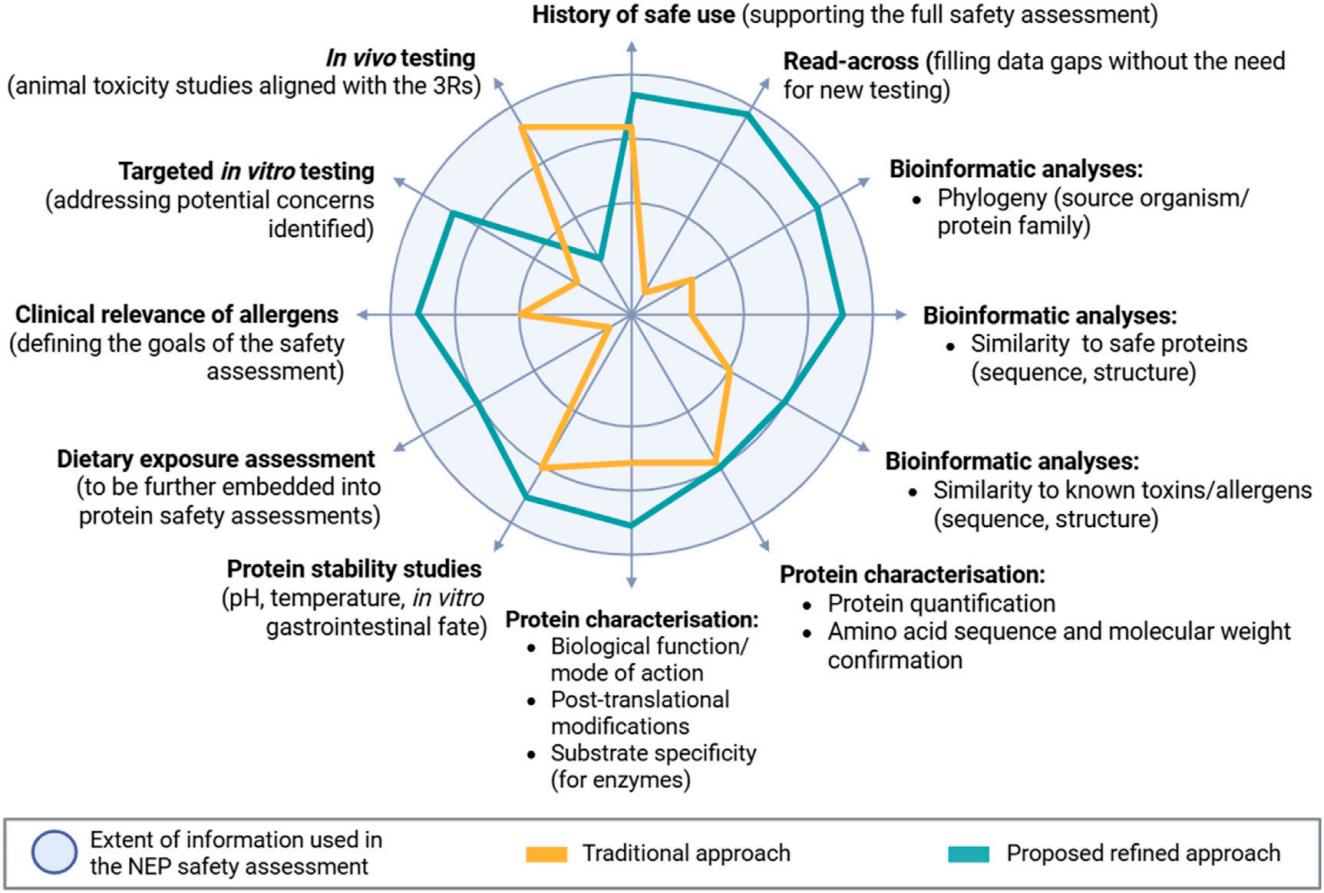
Comparison of the traditional and proposed refined approaches to EFSA’s NEP safety assessment. The circle illustrates the extent of information used in the safety assessment of the NEP. The orange area represents the traditional approach, while the blue depicts the refined proposed approach that EFSA is moving towards. The different sections reflect the components on which the weight-of-evidence approach is based.

The traditional and refined approaches are illustrated in Figure 1. The circle represents the extent of information, and the relative WoE assigned to each component in a NEP safety assessment. The traditional framework (indicated in orange) places significant emphasis on protein stabilities studies, and *in vivo* animal testing, and less weight to HoSU and protein characterisation. The larger area of the circle covered by the refined approach (represented in blue) indicates the integration of more information and lines of evidence into the assessment, thereby reducing reliance on *in vivo* testing. The refined approach gives more value to HoSU, bioinformatic analyses, protein characterisation, protein stability studies, while advocating for reserving animal studies only for cases where a specific hypothesis exists or where critical information is required to assess the safety of the novel protein. Thus, shifting the focus toward other lines of evidence more proportionate to the identified level of potential harm.

For instance, traditional protein characterisation mainly focuses on aspects such as protein quantification, and confirmation of amino acid sequence and molecular weight. On the other hand, the refined approach aims to obtain a deeper understanding of biological function and mode of action of the NEP or substrate specificity in the case of enzymes. Additionally, the traditional approach focuses on similarity to known toxins and allergens, while the refined approach also considers similarity to safe proteins and phylogenetic relationships, using up-to-date available information. In terms of *in vitro* testing, the refined framework recommends integrating gastric and intestinal digestion informing on the fate of the NEP in the gastrointestinal tract and calling for more targeted *in vitro* studies when necessary to address concerns related to general toxicity and potential allergenicity. Finally, dietary exposure, which does not have a prominent role in the current approach, could assume a more central role in the refined framework as recently proposed by EFSA GMO Panel et al. (2025b) and Crevel et al. (2024).

We provide here examples from recent assessments in which EFSA has applied novel scientific evidence and tools such as protein structure analysis, similarity to dietary proteins with a HoSU, clinical relevance of hazard predictions and functional comparability based on mode of action.

## 3 EFSA’s recent assessments following a refined approach

### 3.1 The key role of HoSU and exposure

For NEPs not previously assessed, EFSA and Codex guidelines (EFSA GMO Panel, 2011; Codex Alimentarius, 2003–2009) state that data should be provided on the effect of temperature and pH on the stability of the NEP, its resistance to proteolytic enzymes, like pepsin, using standardised *in vitro* assays and animal testing in cases where HoSU is not documented.

During the assessment of the ZMM28 protein, characterisation data were submitted demonstrating that the ZMM28 protein produced in a GM maize is identical to the endogenous ZMM28 protein naturally present in the GM plant and in most conventional maize varieties. The ZMM28 protein is a MADS-box transcription factor (Castelan-Munoz et al., 2019; Munster et al., 2002) that participates in processes such as photosynthesis, nitrogen assimilation, and growth-regulating hormone signalling. The intended trait derived from its increased and extended expression in this event was potential yield enhancement (Wu et al., 2019). This information was complemented with data supporting a HoSU for ZMM28 protein consumption by both humans and animals, considering exposure to this protein through conventional food and feed products, and no indications of potential adverse effects associated with ZMM28 exposure reported. Consequently, the EFSA GMO Panel deemed studies on the effect of temperature and pH on ZMM28, its *in vitro* degradation by proteolytic enzymes and an animal toxicity study for ZMM28 unnecessary (EFSA GMO Panel et al., 2024b). This approach avoided the generation of data that would not contribute meaningfully to consumer safety.

### 3.2 The relevance of 3D structure analysis and read-across

The assessment of the DGT-28 EPSPS enzyme expressed in a GM maize (EFSA GMO Panel et al., 2025a) illustrates how structural similarity to previously assessed NEPs and/or proteins with a HoSU can render animal studies unnecessary while ensuring a robust safety evaluation.

The DGT-28 EPSPS enzyme confers tolerance to glyphosate-containing herbicides by acting on the shikimic acid pathway, which is involved in the biosynthesis of aromatic amino acids in plants, fungi, and bacteria. Although other EPSPS proteins have been widely used in GM plants and thoroughly assessed by the EFSA GMO Panel, DGT-28 EPSPS belongs to a newly identified Class IV of EPSPS enzymes. This class has been investigated through X-ray crystallography, kinetic analyses, and phylogenetic comparisons across all other classes of EPSPS enzymes. These studies showed that Class IV enzymes form a distinct clade compared to the other three classes and are most closely related to those in Class I (Griffin et al., 2021). This class includes native EPSPS proteins from soybean (*Glycine max)* and maize (*Zea mays*), species widely consumed by humans and animals, and the 2mEPSPS protein, previously evaluated by EFSA GMO Panel et al. (2017b) and EFSA GMO Panel et al. (2020).

The analysis of the protein 3D structure showed that the full-length DGT-28 EPSPS protein exhibited the highest structural similarity to the Class I EPSPS enzyme from *Escherichia coli*, followed by native EPSPS proteins from soybean and maize with a HoSU. Moreover, the active site architecture was found to be highly conserved across all EPSPS enzymes analysed. The amino acid residues involved in substrate binding and catalysis were highly conserved between DGT-28 EPSPS and Class I enzymes, supporting the conclusion that these proteins share the same mode of action.

Based on this information, it was concluded that DGT-28 EPSPS was both functionally and structurally comparable to previously assessed EPSPS variants and to native EPSPS proteins commonly present in the human and animal diet (EFSA GMO Panel et al., 2025a). Thus, the EFSA GMO Panel determined that an animal toxicity study was unnecessary and strongly supported the conclusion that the DGT-28 EPSPS protein does not pose a safety concern for human or animal health (EFSA GMO Panel et al., 2025a), exemplifying EFSA’s shift towards a more proportionate, evidence-based approach to NEP safety assessment.

### 3.3 The expanding role of the mode of action in safety assessment

EFSA’s evaluation of the Vpb4Da2 protein, a vegetative insecticidal protein from *Bacillus thuringiensis* expressed in GM maize (EFSA GMO Panel et al., 2024a; Kouadio et al., 2021), reflects the growing emphasis placed on mode of action and 3D structure in NEPs safety assessment.

Here, bioinformatic analyses revealed that Vpb4Da2 shared significant sequence identity with P13423, the protective antigen (PA) from *Bacillus anthracis*, and with the *Clostridium botulinum* C2-II protein, both of which are components of bacterial toxins (EFSA GMO Panel et al., 2024a; Knapp et al., 2016). Moreover, the GMO Panel determined that the available information on the HoSU was not sufficient to conclude on the food and feed safety of the Vpb4Da2 protein, as the level of exposure to this protein through human and animal consumption had not been established.

The Vpb4Da2 protein belongs to the Bacterial_exotoxin_B family of membrane pore-forming toxins and consists of six structural domains. Domains I–III mediate oligomerisation and pore formation, while domains IV–VI are responsible for receptor binding and specificity. Notably, the structural analysis determined the N-terminal domains (I–III) of Vpb4Da2 shared structural similarity with the PA and C2-II components, whereas the C-terminal domains (IV–VI) differed from the receptor-binding regions of these bacterial toxins, indicating interaction with distinct membrane receptors and corroborating the insect-specific mode of action of Vpb4Da2 (EFSA GMO Panel et al., 2024a; Kouadio et al., 2021). Furthermore, unlike the Vpb4Da2 protein, PA and C2-II proteins are non-toxic subunits that require a specific binary partner to exert their activity (Schleberger et al., 2006; Sherer et al., 2007). This, together with the limited similarity observed between Vpb4Da2 and these bacterial toxins in the receptor-binding regions and other key functional residues, further supports the conclusion that Vpb4Da2 is unlikely to pose a risk to human or animal health.

Considering the available evidence, including protein stability studies, structural data indicating specificity to the target pest, and the fact that *B. anthracis* PA and *C. botulinum* C2-II proteins require additional proteins to exert their toxic activity (Knapp et al., 2016), the EFSA GMO Panel determined that the Vpb4Da2 protein is unlikely to act in the same manner as these bacterial toxins. This conclusion was reinforced by the absence of adverse effects in a 28-day toxicity study.

All in all, the assessment of Vpb4Da2 highlights the growing importance of mode of action and 3D structural analyses in EFSA’s evaluation of NEPs. In earlier assessments, specific experimental evidence was required to investigate potential haemolytic activity when a NEP showed significant sequence identity to bacterial proteins annotated as possible haemolysins (EFSA GMO Panel, 2015). In contrast, the Vpb4Da2 case marks a shift toward a more targeted and informative WoE approach. This evolution reduces reliance on traditional laboratory testing and supports more proportionate assessments aligned with the level of potential harm identified.

### 3.4 The importance of the clinical relevance of allergens in evaluating the potential allergenicity of NEPs

According to Codex guidelines (Codex Alimentarius, 2003–2009) and Regulation (EU) No 503/2013, a sequence alignment showing more than 35% identity over a window of at least 80 amino acids is the minimum threshold that triggers further allergenicity assessment including human sera testing. Nonetheless, this threshold is conservative and assessed on a case-by-case basis by EFSA GMO Panel (2010); EFSA GMO Panel (2017a); EFSA GMO Panel et al. (2022).

This approach is exemplified by EFSA’s evaluation of the Cry14Ab-1 protein expressed in a GM soybean. An initial screen revealed >35% sequence identity between Cry14Ab-1 and an allergen listed in the Compare database (van Ree et al., 2021). As a result, the EFSA GMO Panel requested a detailed evaluation of the alignment (EFSA GMO Panel et al., 2021). Interestingly, this similarity was not identified in assessments by other food safety authorities (ISAAA, 2024).

The EFSA evaluation showed that the 35.4% identity was achieved only by introducing 13 gaps to meet the 80-amino acid alignment requirement. Furthermore, only a three-residue stretch of contiguous identity was present within the aligned region, and the E-value was extremely high (≥99), suggesting the match was likely due to random chance. Only 5 of the 15 amino acids forming a known linear T-cell epitope in Asp f 22 (Oseroff et al., 2012) aligned with Cry14Ab-1, and these matches were discontinuous. Structural comparison between Cry14Ab-1 and the predicted 3D structure of Asp f 22 revealed no meaningful conformational similarity, indicating a very low likelihood of shared epitopes. In addition, full-length sequence alignment yielded only 7% identity between Cry14Ab-1 and Asp f 22, a value far below those observed among fungal enolases known to exhibit IgE cross-reactivity (Lai et al., 2002; Simon-Nobbe et al., 2000).

The Panel also assessed the clinical relevance of Asp f 22 and found that its allergenicity was supported only by *in vitro* IgE binding, with no available data on biological activity (Banerjee and Kurup, 2003; Chaudhary et al., 2010). It is classified as a minor allergen, with specific IgE binding detected in only ∼30% of sera from *Penicillium*-sensitized asthmatic individuals (Lai et al., 2002), and its sensitization appears to occur solely via the respiratory route (Singh et al., 2010). Compounding these uncertainties, the number of relevant human sera available for testing was insufficient to meet Codex recommendations, which call for a minimum of 24 samples when assessing minor allergens (Codex Alimentarius, 2003–2009).

Taken together, these findings indicated that Asp f 22 is of limited clinical relevance. Consequently, the EFSA GMO Panel concluded that additional human serum testing was not required and that the Cry14Ab-1 protein did not pose an allergenicity concern (EFSA GMO Panel et al., 2021).

This case underscores the importance of robust, fit-for-purpose allergen databases and highlights how clinical relevance should be central in evaluating potential allergenic risks (Fernandez et al., 2021; EFSA GMO Panel et al., 2022; EFSA GMO Panel et al., 2025b; Mills et al., 2024). Ultimately, it illustrates EFSA’s commitment to a more transparent, targeted, and science-driven approach to risk assessment.

## 4 Discussion

The safety evaluation of NEPs remains a cornerstone in the risk assessment of GMOs. EFSA applies a comprehensive and structured framework grounded in scientific rigor, transparency, and precaution, with the objective of ensuring a high standard of consumer protection. Nevertheless, there is increasing recognition within EFSA and among global regulatory bodies, that traditional assessment paradigms may not always be proportionate to the actual risk posed by individual NEPs.

In response, EFSA has been refining its methodology, integrating modern scientific tools such as comparative structural biology, clinical relevance assessment of allergenic potential, and evolutionary analysis. These new approaches facilitate a more refined identification of potential hazards, supporting the modernisation of risk assessment without compromising safety assurances. The ongoing refinements aim to: i) support more evidence-based and proportionate risk assessments, ii) align the extent of assessment with the actual potential for harm, and iii) weight prior knowledge and contextual evidence to streamline the evaluations. To this end, the case studies presented in this manuscript illustrate how innovative methodologies, as well as established approaches reconsidered from a new angle, can guide case-by-case decisions, reduce reliance on animal and *in vitro* untargeted studies, and enhance the overall robustness, proportionality and scientific credibility of the risk assessment process.

Implementing a more proportionate approach revealed several new challenges. The concept of proportionality requires clear, commonly understood criteria to ensure consistent application. Variation exists among competent authorities globally in the extent and type of data considered sufficient to support safety conclusions. For example, not all NEPs necessitate the same depth of investigation, particularly when they are structurally and functionally similar to proteins with a well-established HoSU. Yet, the level of evidence considered adequate to conclude on the safety of NEPs may differ depending on the assessor’s interpretation and the regulatory context. EFSA has recently addressed these issues in a dedicated opinion outlining opportunities for future methodological advancements (EFSA GMO Panel et al., 2025b), including a new tiered, proportionate WoE framework for NEP safety assessment, which offers flexibility across a range of scenarios to tailor data requirements on a case-by-case basis (see Figure 3 in EFSA GMO Panel et al., 2025b).

Looking ahead, efforts should focus on further standardising the application of advanced scientific tools, better integrating NAMs into risk assessment. When embedded within a well-defined WoE framework, NAMs methodologies have the potential to enhance the efficiency, consistency, and robustness of NEP safety evaluations, reducing the use of untargeted studies and animal testing in line with the 3Rs. As EFSA and other regulatory bodies continue to modernise their approaches, maintaining the scientific integrity of the risk assessment process remains paramount. At the same time, it is essential to avoid unnecessary data requirements that do not meaningfully contribute to consumer safety. Advancing international collaboration and promoting regulatory convergence will be critical to achieving both scientific credibility and coherence across authorities. Recent initiatives, like the International Medicines Regulators’ Working Group on the 3Rs (European Medicine Agency, 2025), demonstrate how international cooperation can drive regulatory alignment and serve as a model for similar efforts in this field.

Most importantly, international harmonisation efforts should go beyond aligning final risk assessment outcomes. They should also focus on improving the transparency, consistency, and scientific quality of the reasoning sustaining safety assessments. A scientifically valid safety conclusion is only as credible as the evidence narrative that supports it. Therefore, improving the clarity of data interpretation and the justification of conclusions within risk assessments will be essential to maintaining public trust and regulatory alignment across food safety authorities around the world.

## Data Availability

The original contributions presented in the study are included in the article/supplementary material, further inquiries can be directed to the corresponding author.

## Abbreviations

3D, Three-dimensional; 3Rs, Replacement, reduction and refinement of animal testing; Cry, Parasporal crystal; EC, European Commission; EFSA, European Food Safety Authority; EPSPS, 5-Enolpyruvylshikimate-3-phosphate synthase; EU, European Union; GM, Genetically Modified; GMO, Genetically Modified Organisms; GMO Panel, Panel on Genetically Modified Organisms; HoSU, History of safe use; HPPD, Hydroxyphenylpyruvate dioxygenase; IgE, mmunoglobulin E; NAMs, New Approach Methodologies; NEP, Newly Expressed Protein; PA, Protective Antigen; WoE, Weight of evidence.
